# Supplementary material for: Development of a Novel UPLC-MS/MS Method for the Simultaneous Determination of 16 Mycotoxins in Different Tea Categories
Source: Toxins (Basel). 2022 Feb 24;14(3):169. doi: 10.3390/toxins14030169 (PMC8951691; doi:10.3390/toxins14030169)
Supplement: Supplementary file 1 [file toxins-14-00169-s001.zip › toxins-1589311-supplementary.pdf]

Supplementary Materials: Development of a Novel UPLC-MS/MS Method for the Simultaneous Determination of 16 Mycotoxins in Different Tea Categories

Haiyan Zhou, Zheng Yan, Song Yu, Aibo Wu and Na Liu

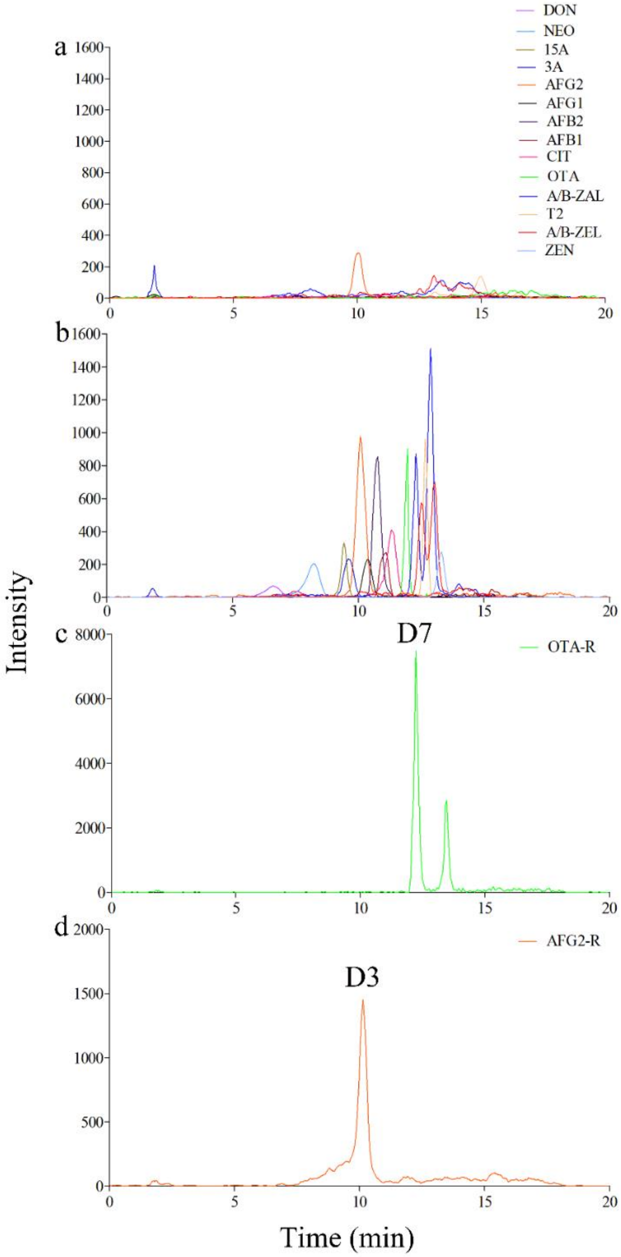

Figure. S1. The chromatogram of blank matrix, bank matrix with mycotoxin standard and mycotoxin in real sample application (R): (a) the chromatogram of blank matrix; (b) the chromatogram of bank matrix with 16

mycotoxin standards; (c) the chromatogram of OTA in real sample (D7); (d) the chromatogram of AFG_2 real sample (D3).

Table S1. Overview of the methodological characteristics including limits of detection, limits of quantification, spiked concentrations (*n* = 6) for various mycotoxins (μg·kg^{-1}).

Analytes	LOD	LOQ	Low	Middle	High
AFB_1	0.03	0.06	0.50	2.50	10.00
AFB_2	0.015	0.03	0.13	0.63	2.50
AFG_1	0.03	0.06	0.50	2.50	10.00
AFG_2	0.03	0.06	0.13	0.63	2.50
ZEN	0.13	0.25	1.00	5.00	20.00
α-ZEL	0.75	1.50	3.00	15.00	60.00
β-ZEL	0.75	1.50	3.00	15.00	60.00
α-ZAL	0.75	1.50	3.00	15.00	60.00
β-ZAL	0.75	1.50	3.00	15.00	60.00
DON	7.50	15.00	30.00	150.00	600.00
15-Ac DON	15.00	30.00	60.00	300.00	1200.00
3-Ac DON	7.50	15.00	30.00	150.00	600.00
OTA	0.125	0.25	3.00	15.00	60.00
NEO	0.75	1.50	3.00	15.00	60.00
T-2	1.50	2.50	3.00	15.00	60.00
CIT	0.06	0.125	10.00	50.00	200.00

Table S2. Occurrence and concentration levels of 16 mycotoxins in pu-erh during different production steps (μg·kg^{-1}).

Analytes	RM	HT	F_1	F_2	F_3	F_4	F_5	QD	BCP	CP
DON	175.51	183.14	226.59	0.00	0.00	0.00	0.00	0.00	0.00	0.00
3-Ac DON	0.00	0.00	0.00	0.00	0.00	0.00	0.00	0.00	0.00	0.00
15-Ac DON	40.24	0.00	557.53	229.67	246.14	174.51	0.00	150.61	63.76	0.00
AFB_1	0.03	0.11	0.09	0.00	0.11	0.04	0.00	0.00	0.00	0.14
AFB_2	0.08	0.13	0.07	0.15	0.13	0.14	0.17	0.00	0.00	0.10
AFG_1	0.42	0.82	3.08	1.74	1.63	1.66	1.55	4.18	1.46	0.86
AFG_2	9.37	12.42	9.72	14.31	10.97	10.75	10.76	5.53	4.53	11.18
ZEN	4.25	4.23	4.77	4.14	4.94	4.46	5.76	4.98	4.02	6.85
α-ZEL	21.13	4.47	10.60	7.30	6.27	7.86	7.56	6.44	6.40	7.03
β-ZEL	49.96	70.47	74.08	66.36	62.83	59.67	59.69	56.16	52.26	72.61
α-ZAL	0.00	14.08	0.00	0.00	0.00	0.00	0.00	0.00	0.00	0.00
β-ZAL	0.00	0.00	0.00	0.00	0.00	0.00	0.00	0.00	0.00	0.00
NEO	0.00	0.00	24.34	18.76	4.98	8.00	3.22	5.53	0.00	0.00
OTA	0.74	0.70	0.38	0.00	0.11	0.18	0.53	0.00	0.00	0.00
T-2	0.00	0.00	11.28	0.00	0.00	0.00	0.00	0.00	0.00	0.00
CIT	8.29	23.22	34.88	48.31	62.92	38.32	34.43	0.00	0.00	0.00
AFs	9.91	13.48	12.96	16.20	12.84	12.59	12.48	9.71	5.99	12.28
ZENs	75.34	93.25	89.46	77.81	74.04	71.99	73.00	67.57	62.68	86.48

RM: raw material; HT: humidifying tea; F_1: first repiling samples; F_2: second repiling samples; F_3: third repiling samples; F_4: fourth repiling samples; F_5: fifth repiling samples; QD: piling-up samples; BCP: semifinished pu-erh; CP: finished pu-erh. The number of samples in each stage of repiling was three, which are taken from the upper, middle, and lower layer.
